# *Aloe vera* (*Aloe barbadensis* miller) gel extract enhances the immune response and tissue regeneration in the zebrafish larvae model

**DOI:** 10.3389/fvets.2026.1852633

**Published:** 2026-08-12

**Authors:** Javiera F. De la Paz Montt, Patricio Yañez-Bailey, Nicolás Zambrano, Priscila García-Castro, Fernando Cruzat, Alejandra Llanos-Rivera, Karen Fehrmann-Cartes

**Affiliations:** 1 Laboratorio de Embriotoxicología e Interacción Desarrollo Ambiente (LEIDA), Facultad de Ciencias Biológicas, Universidad de Concepción, Concepción, Chile; 2 Danio Biotechnologies SpA, Santiago, Chile; 3 Laboratorio de Toxicología Acuática, Departamento de Oceanografía, Facultad de Ciencias Naturales y Oceanográficas, Universidad de Concepción, Concepción, Chile; 4 Laboratorio de Detección Molecular de Microalgas Tóxicas (LDMMTx), Departamento de Oceanografía, Facultad de Ciencias Naturales y Oceanográficas, Universidad de Concepción, Concepción, Chile; 5 Núcleo de Investigación en Producción y Salud de Especies Acuáticas (NIP-SEA), Facultad de Medicina Veterinaria y Agronomía, Universidad de Las Américas, Concepción, Chile

**Keywords:** *aloe vera*, immunostimulant, soybean, wound regeneration, zebrafish

## Abstract

Aquaculture is one of the world’s major food-producing industries; however, intensive farming conditions expose fish to multiple stressors that impair immune function and tissue regeneration, increasing susceptibility to infectious diseases. Therefore, sustainable strategies to enhance wound healing and fish health are needed. This study evaluated the regenerative and immunomodulatory potential of *aloe vera* (*Aloe barbadensis* Miller) gel using zebrafish (*Danio rerio*) larvae subjected to tail tip amputation. *Aloe vera* was administered by immersion and dietary supplementation before and/or after injury. Regenerative and immune responses were assessed by quantifying leukocyte recruitment, analyzing the expression of genes involved in inflammation, oxidative stress, and tissue regeneration, and evaluating larval growth and skin mucus-producing cells. *Aloe vera* enhanced leukocyte recruitment and significantly improved tissue regeneration following injury. These effects were accompanied by modulation of innate immune markers (*il1b, mpx, mpeg1.1,* and *cox-2a*) and antioxidant defense genes (*sod1, sod2,* and *gpx1a*). Dietary supplementation with *aloe vera* also alleviated the detrimental effects of an inflammatory plant protein-based diet on regeneration, promoted larval growth, and increased both the number and size of skin goblet cells. Collectively, these findings demonstrate that *aloe vera* enhances immune function, antioxidant responses, tissue repair, and mucosal protection, supporting its potential as a natural functional additive to improve fish health and welfare in aquaculture.

## 1 Introduction

Fish aquaculture is a fast-growing production sector that provides approximately 20% of animal protein worldwide and has exceeded the supply from wild fisheries (1). However, the progressive intensification of fish aquaculture production systems and climate change have increased the susceptibility of farmed fish to various health disorders. Among these, skin lesions are particularly prevalent in salmon farming and are primarily, but not exclusively, associated with infestation by ectoparasites such *as Caligus rogercresseyi*, a copepod that feeds on mucus, epithelial tissue, and blood. Opportunistic bacterial pathogens can further exacerbate these lesions and promote secondary infections (2–5), causing chronic stress and inflammatory states that can ultimately lead to detrimental consequences, including reduced feed-conversion efficiency, smaller fish, and higher discard rates due to skin damage detected during processing (6, 7).

The immune system contributes to physiological homeostasis through interconnected molecular and cellular defense mechanisms. Within the innate immune system, mucosal surfaces, including the skin, gills, and gastrointestinal tract, constitute the first line of defense against aquatic pathogens (8, 9). When these barriers sustain persistent damage from skin lesions, wounds caused by ectoparasite infestations, or constant cage abrasion, the inflammatory response may become prolonged or dysregulated. Current strategies to control these problems in fish aquaculture rely largely on pharmacological interventions, including antiparasitic treatments and antibiotics. However, these approaches often show limited efficacy, involve high operational costs, and may negatively affect marine ecosystems (10). Furthermore, repeated exposure to these compounds can promote tolerance in target organisms and contribute to the emergence of drug-resistant parasite and bacterial populations, further aggravating the problem. Consequently, novel and more sustainable strategies are urgently needed to enhance the natural defenses of fish, thereby improving animal health and ultimately fish production while minimizing environmental impacts. In this context, the use of natural bioactive compounds represents a promising alternative. Extracts derived from *aloe vera* (*Aloe barbadensis* Miller; AV) have attracted increasing attention because of their wide range of biological activities. Therefore, it is important to further investigate the biological responses triggered by AV when administered either as a dietary supplement or as a bioactive compound in the culture medium (11).

*Aloe vera* is widely distributed in arid and semiarid regions worldwide and has long been recognized for its diverse medicinal properties. The leaf contains an inner gel composed of approximately 98% water and 2% dry matter (12), including more than 75 biologically active compounds, such as polysaccharides (including acemannans and fructans), vitamins (A, C, and E), minerals (Ca and Mg), essential amino acids, phospholipids, and organic acids (13). Numerous pharmacological applications have been described in humans, and although different plant components exhibit distinct bioactivities, many therapeutic effects have been attributed specifically to the inner gel fraction (14).

Zebrafish (*Danio rerio*) is a well-established vertebrate model for studying tissue regeneration because of its remarkable ability to regenerate structures such as the caudal fin and tail tip (15, 16). Although adult caudal fin amputation is the most widely used model, early zebrafish larvae [2–5 days post-fertilization (dpf)] have gained increasing attention because they complete regeneration within only 2–6 days, providing a rapid and efficient system for investigating regenerative mechanisms (17, 18).

Epimorphic regeneration of the zebrafish caudal appendage involves three coordinated stages: wound healing, blastema formation, and regenerative outgrowth. The initial wound-healing phase is characterized by reactive oxygen species (ROS) production and acute inflammatory cell recruitment, primarily neutrophils, followed by macrophages. Subsequently, proliferative progenitor cells accumulate to form the blastema, leading to tissue growth and differentiation that restore the original structure (19).

In addition to its regenerative capacity, zebrafish larvae offer several experimental advantages, including optical transparency, well-characterized genetics, and the availability of transgenic reporter lines. Their small size also allows bioactive compounds to be administered by immersion or dietary supplementation, making this model a rapid and cost-effective platform for screening functional compounds with potential applications in aquaculture before validation in commercially important species such as salmonids (20, 21).

The objective of the present study was to evaluate tissue regenerative capacity and the associated immune and antioxidant responses to a controlled wound in zebrafish larvae exposed to whole *aloe vera* gel. For this purpose, a tail-tip amputation assay was performed using the transgenic line Tg(*lysC: D*sRED2), which expresses the red fluorescent protein DsRED2 under the control of the lysozyme C promoter (22) and labels leukocytes, predominantly neutrophils, which represent nearly 100% of the fluorescent cells from approximately 3 dpf onward (23). This model enables *in vivo* visualization and quantification of leukocyte recruitment to the injury site following tissue damage, primarily neutrophils and a few macrophages, as well as measurement of regrowth of the amputated area as an indicator of regenerative capacity (24). Because immune cell counts were conducted from 3 dpf onward, only neutrophils are discussed here. In addition, the expression of key immune and stress-response genes specifically in the tail region, the number and size of mucus-producing goblet cells, which contribute to innate immunity in the skin of the larval trunk region, and the overall larval growth were assessed.

## 2 Materials and methods

### 2.1 Animals

Zebrafish were maintained and raised according to the standard protocols described by Westerfield (25). All protocols and procedures were approved by the Ethics, Bioethics, and Biosafety Committee of the Vice-Rector’s Office for Research and Development at the University of Concepción (project ID20I10078). Adult wild-type Tab5 and transgenic Tg(*lys*C: DsRED2) zebrafish strains were maintained at 26–28 °C in fish water (reverse-osmosis water supplemented with Instant Ocean salt to reach a conductivity of 500–800 μS/cm and equilibrated to pH 7.2 ± 0.2 with sodium bicarbonate) and fed twice daily with GEMMA Micro 500 (Skretting, Westbrook, ME, United States). Embryos were obtained in spawning boxes. At 60 min after the light was turned on, eggs were collected randomly, rinsed with E3 embryo medium (5 mM NaCl, 0.17 mM KCl, 0.33 mM CaCl_2_, and 0.33 mM MgSO_4_, equilibrated to pH 7.2), and maintained at 28 ± 0.5 °C in Petri dishes in a cooled incubator (FOC 215-E, VELP Scientifica, Italy) under a daily photoperiod of 14 h light/10 h dark. Dishes were cleaned daily, the embryo medium was renewed, and dead or abnormal embryos were removed. Embryonic and larval ages are expressed in hours or days post-fertilization (hpf or dpf), according to Kimmel et al. (26).

### 2.2 Tail tip amputation protocol

**Figure 1:**
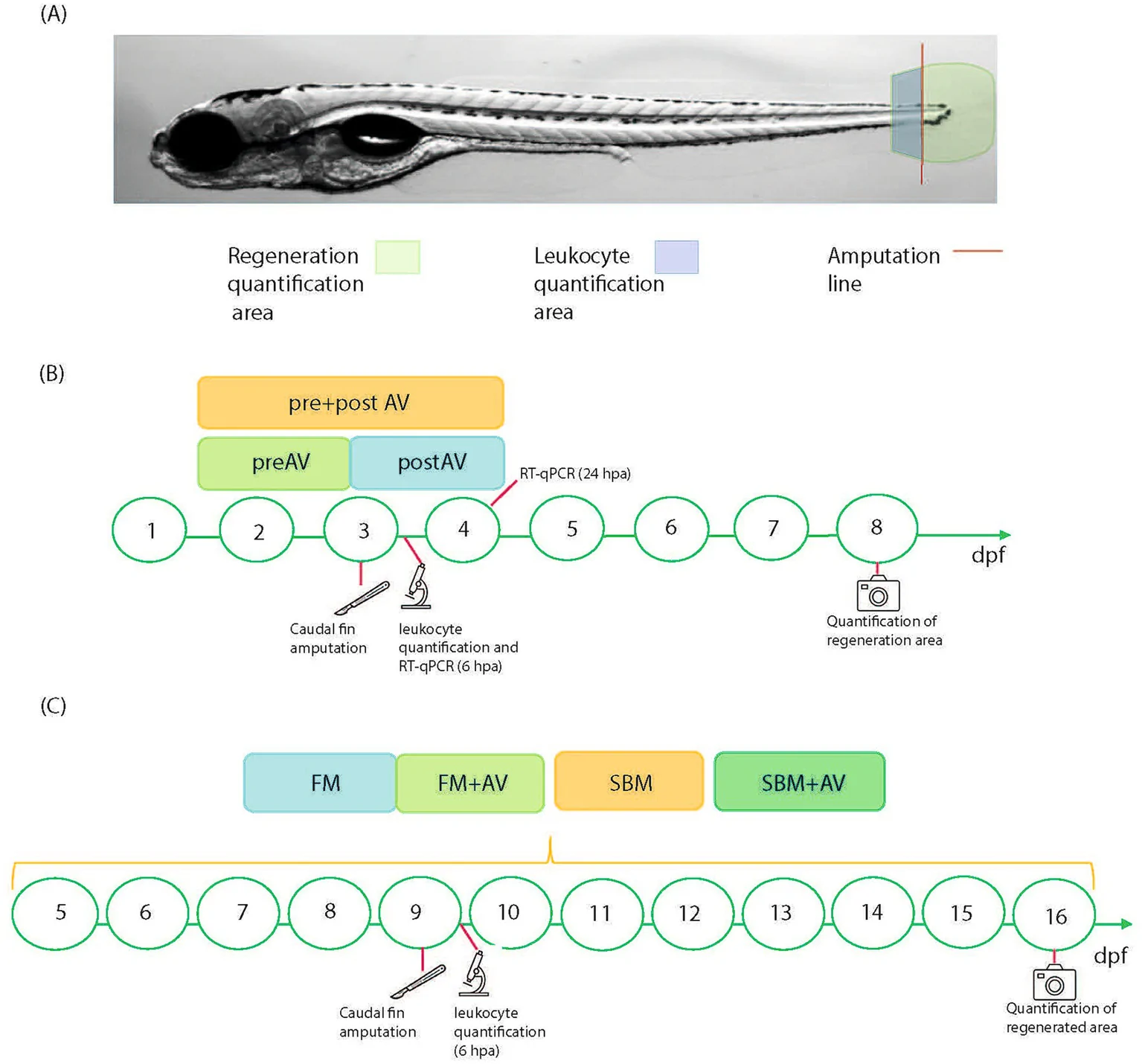
Experimental strategy. **(A)** Caudal fin amputation: An image of a 5 dpf larva shows the anatomical areas relevant to this study. The red line marks the amputation plane, the green line and region indicate the area used to quantify regeneration, and the blue line and region indicate the area used to quantify neutrophils in Tg(*lys*C: DsRED2) larvae. **(B)** Timeline of the immersion experiments. The upper colored boxes indicate the different AV-exposure periods in the experimental groups relative to amputation at 3 dpf. **(C)** Timeline of the feeding experiments. The colored boxes indicate the different dietary treatments administered over 10 days (5–16 dpf). AV, *Aloe vera*; dpf, days post-fertilization; hpa, hours post-amputation.

Zebrafish Tg(*lysC*: DsRED2) larvae at 3 and 9 dpf, used for the immersion and feeding treatments, respectively, were anesthetized with 0.04% MS-222 (Sigma-Aldrich), mounted laterally on a clean glass slide, and the tips of the caudal fins and notochords (tail tips) were amputated with a sterile No. 11 surgical blade, according to Elks et al. (27). The ventral limit of the caudal pigment gap in the tail was used as a reference for the amputation plane and subsequent regeneration-area quantification (Figure 1A), according to Morales et al. (28). The larvae were then immediately and carefully washed with E3 medium to allow recovery and were reincubated. Larvae at 3 dpf were placed in 6 mL of E3 medium in a six-well multiplate (six groups of 10 larvae), whereas larvae at 9 dpf were placed in Tecniplast^®^ polycarbonate breeding tanks containing 250 mL of fish-farming system water (60 larvae per tank; duplicate tanks).

### 2.3 *Aloe vera* gel preparation and incubation

The AV gel was obtained from the leaves of 3-month-old *Aloe barbadensis* Miller plants cultivated in the Coquimbo Region (Region IV), Chile. These plants received 2,000 mL of water per plant every 15 days. Detailed information on the composition of bioactive compounds, such as total sugars, soluble sugars, fructans, acemannans, mannose, glucose, and total protein, is available in Salinas et al. (29) and Quezada et al. (30) because the plants used in this study were grown at the same location under the same cultivation conditions. Aloe gel was liquefied and lyophilized as described by Fehrmann-Cartes et al. (31). For the immersion experiments, a concentrated stock solution of AV powder in Milli-Q water (10 g/L) was prepared and stored at −20 °C until use.

### 2.4 Immersion exposure for larval treatment

For AV administration, larvae were exposed directly to AV gel diluted in E3 medium (0.1 g/L) under three exposure regimens (1): the preAV group, in which 2 dpf larvae were exposed for 24 h until amputation (2); the pre + postAV group, in which 2 dpf larvae were exposed from 24 h before until 24 h after amputation; and (3) the postAV group, in which 3 dpf larvae were exposed immediately after amputation for 24 h. A fourth control group was incubated solely in E3 medium. At least 30 larvae were used for each treatment in triplicate (Figure 1B).

### 2.5 Diet formulation and feeding strategy for oral AV administration

Two basal diets were formulated and prepared using an extruder to contain either fishmeal as the primary protein source (FM; non-inflammatory diet) or 50% soybean meal (SBM; inflammatory diet) as a partial fishmeal replacement (Table 1). One batch of each diet was supplemented with AV at 0.8 g/kg (FM + AV and SBM + AV), which was incorporated during the post-extrusion oiling process. All diets (Figure 1C) were supplemented with a commercial DSM vitamin and mineral premix and formulated to be isoenergetic and isonitrogenous. The diets were prepared as described by Fehrmann-Cartes et al. (31). They were subsequently crumbled, screened to the appropriate particle size (75 μm), and stored at −20 °C until use in the feeding trials. Feeding was performed as follows: groups of 60 larvae at 5 dpf were fed twice daily for 12 consecutive days, with daily renewal of the fish-farming water (see section 2.1) and transfer to a clean tank.

**Table 1:** Ingredients and nutrient composition of experimental diets.

Ingredients/diets (g/kg)	FM	SBM	FM + AV	SBM + AV
Fish meal	610	250	610	250
Soybean meal	0	500	0	500
Wheat grain meal	255	115	255	115
Starch	45	45	45	45
Fish oil	30	60	30	60
Vitamin/mineral mix *	30	30	30	30
Cellulose	30	0	30	0
Total	1,000	1,000	1,000	1,000
AV			0.8	0.8
Analytical composition (dry base, %)				
Dry matter	93.92	94.41	94.90	94.69
Proteins	50.35	50.37	50.85	50.23
Ether extract	8.24	8.39	7.48	8.16
Fiber	5.02	3.04	3.36	4.70
No nitrogenous extract	26.34	29.44	28.56	26.95
Ash	10.06	8.76	8.74	9.97
Gross energy (MJ/kg)	19.70	20.30	20.08	19.74

*Vitamin/mineral premix (DSM), supplied per kilogram of feed: 6.66 IU retinol; 3.33 IU cholecalciferol; 336.67 mg tocopherol; 16.67 mg menadione; 20.00 mg thiamine; 30.00 mg riboflavin; 202.00 mg niacin; 25.00 mg pyridoxine; 66.67 mg pantothenic acid; 0.03 mg cyanocobalamin; 336.67 mg ascorbic acid; 11.67 mg folic acid; 0.67 mg biotin; 7.17 mg Cu; 333.35 mg myo-inositol; 95.00 mg Fe; 59.17 mg Mn; 0.50 mg Co; 5.00 mg I; 179.17 mg Zn; 0.65 mg Se. AV, *Aloe vera*; FM, fishmeal; SBM, soybean meal.

### 2.6 Quantification of neutrophil recruitment to the wound area *in vivo*

Using the Tg(*lys*C: DsRED2) reporter line to identify neutrophils, the number of inflammatory cells was quantified *in vivo* and in real time between 5 and 6 hpa in the tail region anterior to the amputation plane (approximately 150 μm). For this purpose, larvae were placed on a clean glass slide in a drop of culture medium containing 0.04% MS-222 and observed laterally using an inverted epifluorescence microscope (Nikon Eclipse Ts2) at total magnifications of 100 × and 200 × .

### 2.7 Quantification of the regenerated tail and caudal fin area

The total regenerated area was measured at 3, 5, and 7 days post-amputation (dpa), according to the experimental strategy used (administration by immersion or feeding), corresponding to 6, 8, and 16 dpf, respectively. Larvae were anesthetized as described above and immobilized in a small glass Petri dish with a thin layer of 4% methylcellulose to capture images of the caudal fin and tail of live larvae using a bright-field stereomicroscope (Zeiss Stemi 305) at 40 × total magnification. Images were analyzed using ImageJ software and its area-measurement tool. The regenerated area was measured from a line perpendicular to the anteroposterior axis, extending from the anterior limit of the ventral pigment gap in the tail to the contour of the regenerated area at the posterior limit of the caudal tissue.

### 2.8 RNA extraction and RT-qPCR

After exposure, tissue samples were obtained by cutting the tails of 30 larvae per group, immediately posterior to the anus, from anesthetized 3 dpf larvae at 6 and 24 hpa. The samples were immediately stored in 200 μL of RNA Lock Reagent (Omega Bio-tek, Inc., Norcross, GA, United States) at −20 °C until extraction. RNA was extracted exclusively from the larval tails using the E. Z. N. A. Total RNA Kit I (Omega Bio-tek, Inc., Norcross, GA, United States), according to the manufacturer’s recommendations. After extraction, the samples were digested with DNase RQ1 (Promega Corp., Madison, WI, United States), according to the manufacturer’s instructions. RNA concentration and quality were verified by measuring the 260/280-nm ratio using a NanoDrop 1,000 spectrophotometer (Thermo Fisher Scientific, Inc., Waltham, MA, United States).

First-strand cDNA synthesis was performed using a commercial kit (AffinityScript QPCR cDNA Synthesis Kit, Agilent Technologies, Inc., Cedar Creek, TX, United States), according to the manufacturer’s instructions. Reverse-transcription quantitative polymerase chain reaction (RT-qPCR) was performed using an AriaMX G8830A Real-Time PCR Detection System (Agilent Technologies, Inc., Santa Clara, CA, United States). Three genes related to oxidative metabolism (*gpx, sod-1*, and *sod-2*), three genes related to early development and organogenesis (*shh-a, bmp4*, and *fgf20a*), and five genes related to immune and inflammatory responses (*mpx, mpeg1, cox-2a, il-1b, and il-10*) were selected. The *18S* gene was selected as the internal reference gene for data normalization. The primers for these genes are listed in Table 2.

**Table 2:** Primer sequences for the genes tested in the present study.

Gene ID	Forward primer	Reverse primer	GenBank accession No.
*mpx*	gtcggtgcattcaaccagat	atcagtccacggagcacagg	XM_021479113.1
*mpeg1*	cccaccaagtgaaagagg	gtgtttgattgttttcaatgg	NM_212737.1
*cox2a*	actacccctgagcttctcaca	gatgctgttgatgatatcccagattg	NM_153657.1
*il1b*	catttgcaggccgtcaca	ggacatgctgaagcgcactt	NM_212844.2
*il10*	atttgtggagggctttcctt	agagctgttggcagaatggt	NM_001020785.2
*sod1*	aggtgactggtgaaattactgg	gtctcacactatcggttggc	NM_131294.1
*sod2*	ccggactatgttaaagccatct	acactcggttgctctcttttctct	NM_199976.1
*gpx*	aaggagaagcttcctcagcc	agatgtcattcctgcacacg	NM_001007281.2
*shh-a*	aaccactgaacacggacctc	ctccagtttggctccttcag	NM_131063.3
*bmp4*	aaaacgctctaatcatggcct	acgtcgctgaaatccacatac	NM_131342.2
*fgf20a*	gggtctcatttcgtcctcac	acggaggatccctttgagat	NM_001037103.2
*18 s*	agggacaagtggcgttcagc	gcagggtaggcacacgttga	FJ_915075.1

Amplification was performed in eight-well PCR tube strips (Azenta Life Sciences, Inc., Irlam, Manchester, United Kingdom) in a volume of 10 μL containing 5 μL of Takyon ROX SYBR 2X MasterMix dTTP Blue (Eurogentec, Seraing, Wallonia, Belgium), 2 μL of each primer at 2 μM, and 1 μL of cDNA sample. All reactions were performed in quadruplicate. The amplification conditions were as follows: a predenaturation step at 95 °C for 5 min; 40 cycles of 20 s at 95 °C, 30 s at 55 °C (50 °C for the mpeg1 gene), and 30 s at 72 °C; and a final melting-curve analysis to determine reaction specificity. Standard curves were obtained for each primer using at least five dilutions of a cDNA mixture for all analyzed primers. Real-time PCR efficiency (E) was calculated from each standard curve according to the equation E = 10(−1/slope), and the curves were visualized using Agilent AriaMX 1.7.1 software. Relative gene-expression levels were calculated using the 2-ΔΔCT method described by Livak and Schmittgen (32).

### 2.9 Evaluation of larval growth and skin goblet cells

Larval length was measured in live 16 dpf larvae after the 10-day feeding treatment. Individuals were anesthetized and photographed in lateral view while mounted in 4% methylcellulose. Low-magnification images were acquired to quantify larval length from the end of the otic vesicle to the end of the notochord. To detect skin goblet cells, whole larvae were stained with Alcian Blue 8XG (Sigma-Aldrich, No. 05500). Briefly, larvae were euthanized with an overdose of MS-222, fixed for 24 h in 4% paraformaldehyde, and rinsed with PBST. Pigments were then removed with a freshly prepared solution of 3% H2O2/1% KOH for 10 min. The larvae were rinsed again with PBST and stained overnight in an acidic solution (0.1% Alcian Blue/70% ethanol/0.1% HCl) to detect acidic mucins from goblet cells. To remove excess dye, larvae were rinsed in 70% ethanol and cleared in acidic ethanol (70% ethanol/0.1% HCl). Images of the trunk region were captured, and a fixed area delimited by the beginning of the midgut and the anus was used for cell-number quantification and measurements. In both cases, larval images were acquired using an EUREKAM 3.0 PLUS camera coupled to a microscope with 10 × and 40 × objectives (BEL, BIO2T).

### 2.10 *Statistical* analysis

Statistical analysis and data visualization were performed using GraphPad Prism 10 software (San Diego, CA, United States). Shapiro–Wilk and Bartlett’s tests were performed to assess data distribution and homoscedasticity, respectively. For normally distributed data with equal variances, one-way ANOVA followed by Tukey’s *post hoc* test was used; otherwise, the Kruskal–Wallis test followed by Dunn’s multiple-comparison test was performed. At least 60 larvae were included in each experiment, and triplicate groups were used to measure more than one parameter in accordance with the 3Rs principle. Data are presented as means and standard deviations. Statistical significance was set at *p* < 0.05.

## 3 Results

### 3.1 Immersion exposure to AV increases neutrophil recruitment and tissue regeneration

**Figure 2:**
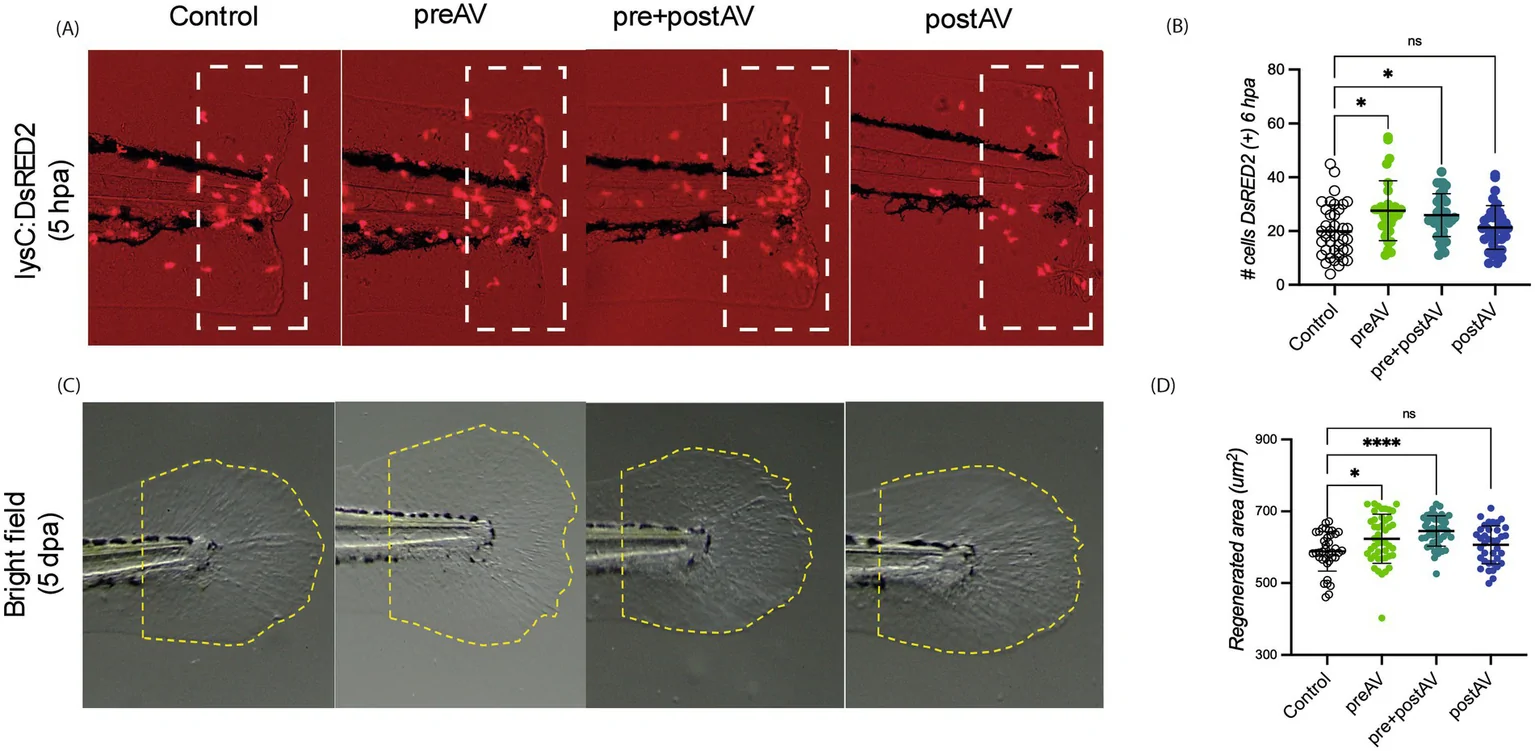
AV exposure by immersion increases neutrophil recruitment and the regenerated caudal fin area. **(A)** Recruitment of neutrophils (red DsRED+ cells) in the control and treatment groups at the amputation site. **(B)** Quantification of neutrophils in 3 dpf larvae at 6 hpa (*N* = 29–37). **(C)** Tail and caudal fin of amputated larvae after 5 days of regeneration. **(D)** Quantification of the regenerated area at 5 dpa (*N* = 37–45). All scatter plots show the mean ± *SD*. Kruskal–Wallis test with Dunn’s multiple-comparison test. *p < 0.05; ****p < 0.0001; ns, not significant. AV, *Aloe vera*; dpf, days post-fertilization; hpa, hours post-amputation; dpa, days post-amputation; SD, standard deviation.

To assess the effect of AV on the inflammatory response to tail-tip excision, we used Tg(*lys*C: DsRED2) larvae at 3 dpf. At 6 hpa, neutrophil recruitment to the amputated area was greater in both groups incubated with AV before injury (preAV, *p* < 0.05; pre + postAV, *p* < 0.05) than in the control group (Figures 2A, B). However, the differences disappeared by 24 hpa, indicating resolution of inflammation in all groups and suggesting that pre-amputation treatment with AV may have an immunostimulatory effect. The tissue-regeneration area was quantified at 3 and 5 dpa. No differences were observed until day 5, when the preAV (*p* < 0.05) and pre + postAV (*p* < 0.0001) groups showed a greater regenerated tissue area than the control group (Figures 2C,D). These results show that AV administered in the culture medium can enhance the innate cellular response and increase tissue regeneration after tail-tip amputation in zebrafish larvae.

### 3.2 AV upregulates key genes involved in regeneration, innate immunity, and the antioxidant response

Next, we sought to determine whether the pro-regenerative and immunostimulatory effects of AV observed after larval tail-tip amputation were reflected in transcriptional changes, specifically in the larval tail tissue adjacent to the wound. For this purpose, we quantified the expression of genes involved in tissue regeneration (*fgf-20, bmp4,* and *shh-a*) and the innate immune response (*mpx, mpeg1, il-1b,* and *cox-2a)*. In addition, because the antioxidant effects of this plant are widely recognized, we analyzed the expression of the main enzymes that regulate oxidative stress in larvae (*gpx, sod-1*, and *sod-2*) to determine whether AV immersion treatment activates the redox-protection response in the larval tail.

**Figure 3:**
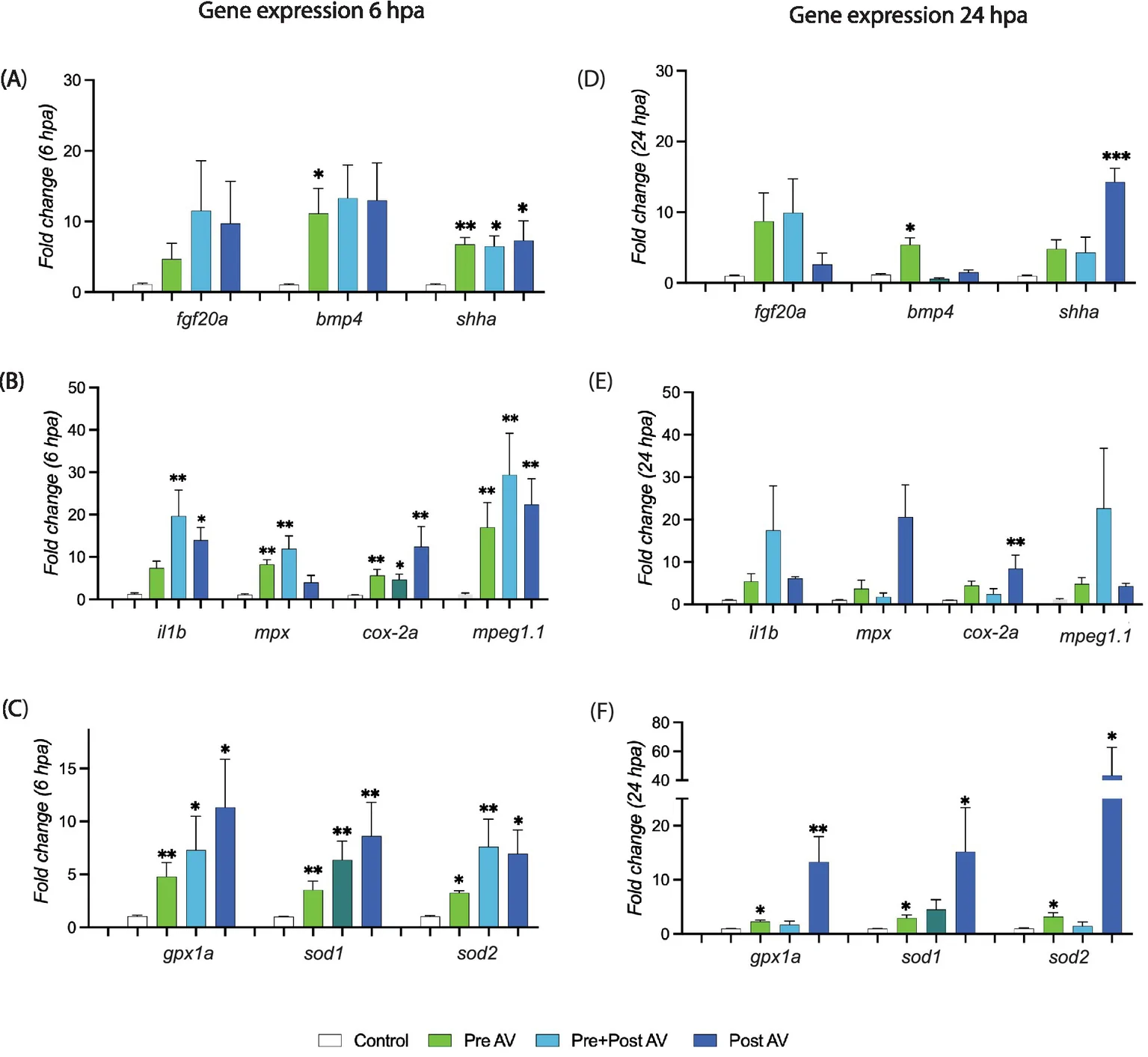
AV exposure by immersion modifies mRNA expression in the tails of amputated larvae. mRNA fold changes in the caudal tissue of larvae at **(A–C)** 6 hpa and **(D–F)** 24 hpa. Average gene-expression levels from three experiments show increased expression of genes associated with regeneration **(A,D)**, immunity **(B,E)**, and the oxidative stress response **(C,F)** in the tails of AV-treated larvae. The data were normalized to the housekeeping gene *18S* and compared with those obtained from amputated control larvae. Kruskal–Wallis test (*p < 0.05, **p < 0.01, ***p < 0.001). AV, *Aloe vera*; mRNA, messenger RNA; hpa, hours post-amputation.

At 6 hpa (Figures 3A–C), the transcription levels of all genes associated with regeneration, immunity, and antioxidant responses were increased in the groups treated with AV before injury (preAV and pre + postAV) and, as expected, returned to control levels in most cases after 24 h. The exceptions were the *bmp4, gpx, sod-1*, and *sod-2* genes in the preAV group, which remained upregulated 1 day after amputation. Additionally, the group treated with AV immediately after injury (postAV) maintained higher expression of *shh-a*, *cox2-a*, and all antioxidant enzymes than the control group at 24 hpa (Figures 3D–F), indicating that this significant increase in expression was directly related to AV exposure. The *fgf-20* expression level was slightly increased in the pre + postAV group at 6 and 24 hpa; however, no significant differences from the amputated control were observed (see the figure legend for *p*-values).

### 3.3 Impact of AV inclusion in zebrafish feed

To determine whether AV could be beneficial under multistressor conditions commonly faced by farmed fish, we evaluated orally administered AV by including whole-leaf gel powder in the diet, a formulation previously reported to have anti-inflammatory properties in zebrafish larvae (31).

**Figure 4:**
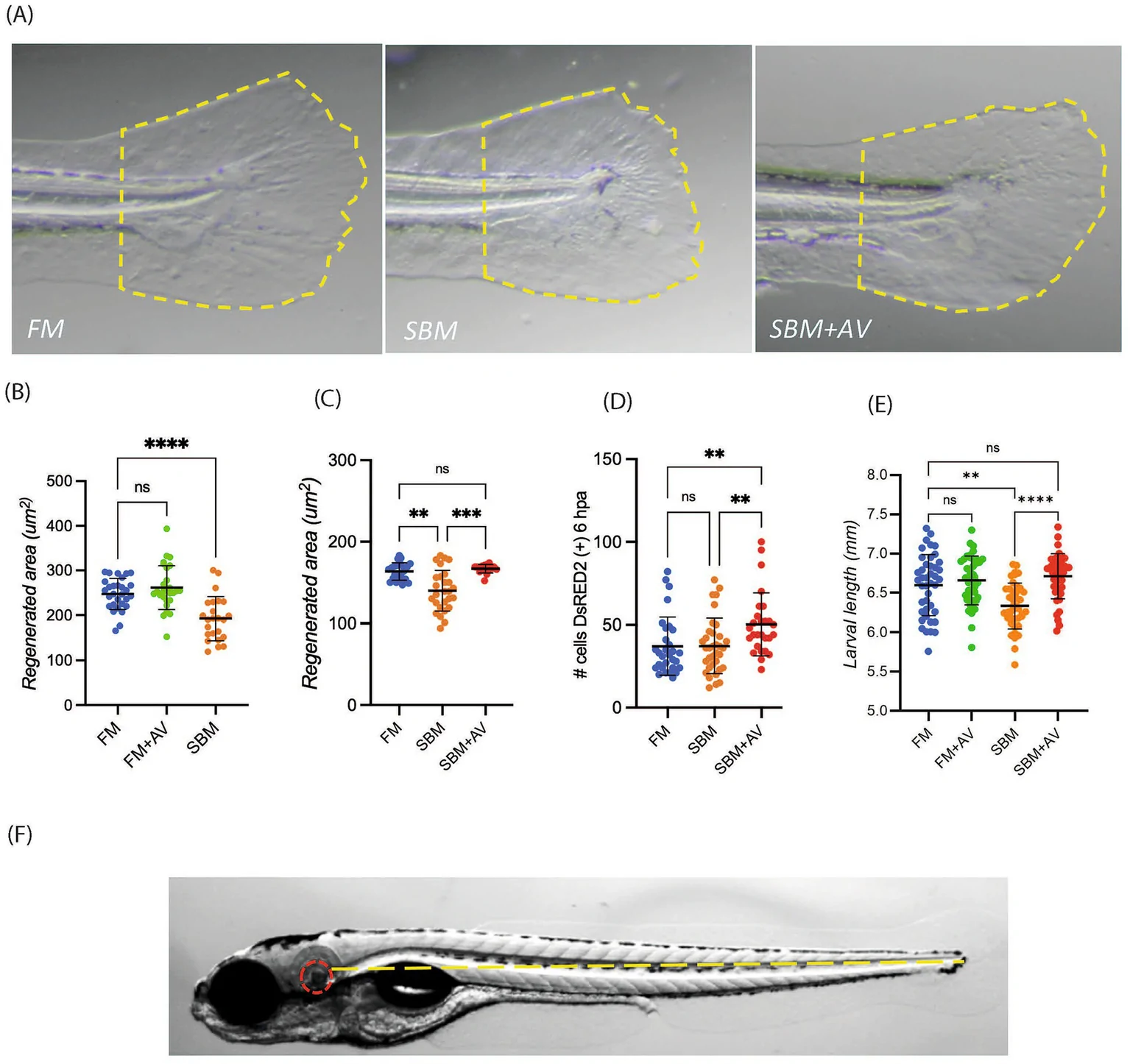
Dietary AV rescues the negative effects of SBM on regeneration and growth. **(A)** Representative images of the caudal regeneration area after 7 days of the feeding treatments. **(B,C)** Quantification of the regenerated area in fed individuals at 7 dpa (*N* = 16–29). **(D)** Neutrophils in the wound area at 6 hpa (*N* = 26–35). **(E)** Larval length after 12 days of feeding. **(F)** Lateral view of a 16 dpf control larva; the yellow dotted line shows the notochord length, and the dotted circle marks the otic vesicle. **(B,E)** One-way ANOVA with Tukey’s multiple-comparison test. **(C,D)** Kruskal–Wallis test with Dunn’s multiple-comparison test. All scatter plots show the mean ± SD. **p < 0.01; ***p < 0.001; ****p < 0.0001; ns, not significant. AV, *Aloe vera*; SBM, soybean meal; dpa, days post-amputation; hpa, hours post-amputation; dpf, days post-fertilization; ANOVA, analysis of variance; SD, standard deviation.

We fed 5 dpf larvae a non-inflammatory fishmeal (FM) diet or an inflammatory soybean meal (SBM) diet (21) for 12 days. After 5 days of feeding, the tail tips were amputated in both groups, and their regenerated areas were evaluated at 5 and 7 dpa. No differences in regenerated tissue area were observed at 5 dpa. However, at 7 dpa, the SBM-fed group showed a smaller caudal tissue area than the FM-fed group (Figures 4A,B) (*p* < 0.01; 0.0001), indicating that SBM negatively affected regeneration after caudal fin amputation and could therefore be used to assess whether orally administered AV counteracts this additional challenge. Based on this result, only regeneration at 7 dpa was assessed in the subsequent experiments.

The data suggest that the SBM diet alone slowed the regenerative process, whereas AV inclusion in the control FM diet (FM + AV) did not further enhance or accelerate regeneration (Figure 4B). When the effect of including AV in the SBM diet (SBM + AV) was tested, tissue growth recovered; no differences in regenerated tissue area were detected between the SBM + AV and control FM groups (Figure 4A, C; *p* < 0.001). Moreover, although neutrophil recruitment to the wound area did not differ between the SBM and FM groups, the SBM + AV diet produced a marked increase in neutrophil presence compared with the SBM and FM diets (Figure 4D) (*p* < 0.001).

Because the effects on regenerated tissue became more evident during the later stages of the process with both AV-administration approaches, we examined whether AV affected overall larval growth by measuring body length after the feeding trials (Figure 4F). The data show that animals fed SBM were smaller than control animals fed FM; interestingly, this effect was reversed by including AV in the SBM diet (Figure 4E) (*p* < 0.001 and *p* < 0.0001, respectively).

### 3.4 Dietary AV increases the number and size of skin mucus-producing cells in zebrafish larvae

**Figure 5:**
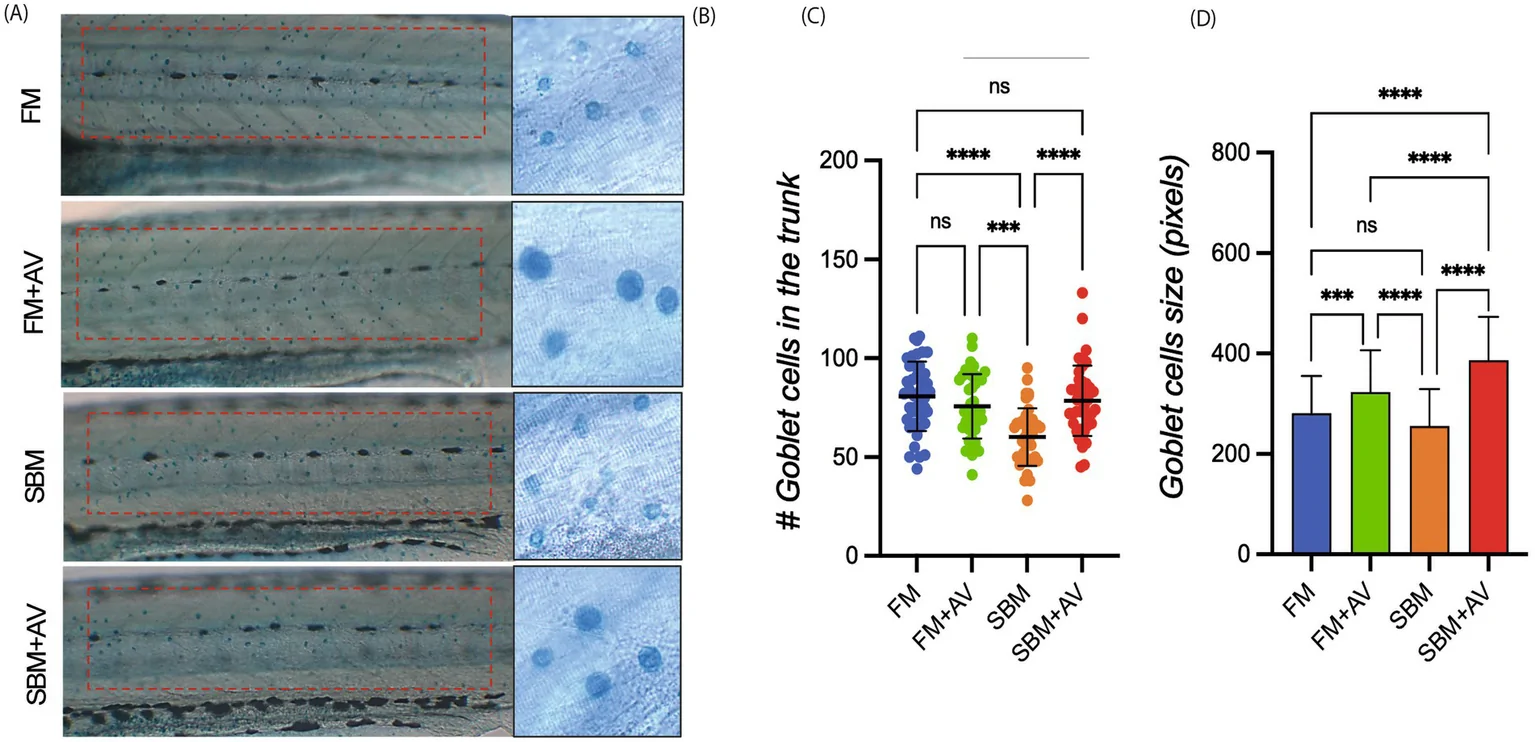
Skin Goblet cells in whole mount zebrafish 16 dpf larvae stained with Alcian blue. **(A)** trunk area and Goblet cells **(B)** of larvae fed for 12 days with the experimental diets. Dotted red box mark the quantification area. **(C)** Number and **(D)** size of Goblet cells in the larval trunk skin. **(C-D)** Graph shows mean ± *SD*. **(C)** One-way ANOVA with Tukey multiple comparison, *N* = 42. **(D)** Kruskall-Wallis test with Dunn multiple comparison, *N* = 15; 10 cells/larva. ***p < 0.001; ****p < 0.0001; ns, no significant.

Finally, considering the importance of skin mucus as a mechanical barrier and the protective and reparative effects that AV showed in the SBM treatment group after tissue damage, we examined whether these experimental diets altered the number and size of mucus-producing cells (goblet cells) in the larval skin as an estimate of mucus production at the skin surface. Previous reports showed that SBM increased mucus production in the *Salmo salar* intestine, whereas AV decreased it (33); however, our results in larval skin showed the opposite. The SBM diet decreased the number of goblet cells in the skin (*p* < 0.0001), whereas AV addition not only reversed this effect when coadministered with SBM (SBM + AV, *p* < 0.0001) but also significantly increased cell size when included in the SBM and FM diets (SBM + AV, *p* < 0.0001; FM + AV, *p* < 0.001) (Figures 5A–D). This result suggests that orally administered AV induces skin mucus production in fish.

## 4 Discussion

The gel of *Aloe vera* leaves contains numerous bioactive compounds that confer a wide range of biological activities. In mammalian models, its hypoglycemic, hypolipidemic, proliferative, antioxidant, and immunostimulatory properties have been widely documented, supporting the traditional medicinal use of AV by different cultures for the treatment of gastric disorders, wound healing, and other conditions (13, 34–36).

Tissue repair following injury represents a major health concern not only in humans but also in animals, particularly in the aquaculture industry. Chronic skin wounds compromise animal health and welfare and negatively affect productivity. Moreover, they often lead to increased use of drugs and biocides to control infections, thereby contributing to environmental contamination. Several external factors, including glucocorticoids, microbial pathogens, undernutrition, environmental toxins, and water temperature, can interfere with or delay wound healing and tissue regeneration in fish (15, 37–39). These factors are particularly relevant in aquaculture systems, where animals are frequently exposed to stressful conditions such as high stocking densities and suboptimal water quality, which are compounded by climate change. Together, these factors facilitate the proliferation of pathogens and parasites capable of damaging epithelial tissues.

Considering this context, the present study aimed to determine whether the therapeutic properties of AV gel could be exploited to improve immune function, antioxidant responses, and tissue regeneration in fish following injury. To address this question, we used the zebrafish larval model and the well-established tail tip amputation assay. This model shares key molecular and cellular mechanisms with adult caudal fin epimorphic regeneration, where muscle, vascular, nervous, and epithelial tissues are lost and subsequently regenerated. In larvae, substantial tissue regrowth occurs within 3 days at 28 °C, with near-complete structural restoration observed by 6 days post-amputation (18).

### 4.1 *Aloe vera* upregulates key genes involved in regeneration, including immune and antioxidant pathways

Regeneration is a complex biological process involving multiple genetic pathways and regulatory networks that are also active during embryonic development. In zebrafish caudal fin regeneration, the coordinated proliferation and differentiation of blastema cells require activation and crosstalk among the bone morphogenetic protein (BMP), fibroblast growth factor (FGF), and Hedgehog (HH) signaling pathways. Additionally, the early production of reactive oxygen species (ROS) during the first 24 h after injury plays a critical role in initiating the regenerative program. Experimental inhibition of any of these pathways severely impairs regeneration (40–45). Although regeneration is primarily governed by these molecular mechanisms, environmental factors such as water temperature have also been shown to influence regenerative outcomes by modulating metabolic activity, cell proliferation, and the rate of tissue regrowth in zebrafish.

FGF signaling regulates the cell proliferation and growth rate of regenerating tissues during zebrafish caudal fin regeneration (46). Expression of *fgf20* is induced as early as 1 hpa, with peaks at 6 and 24 hpa in adult fish (41). During this period, tight regulation of ROS production, particularly hydrogen peroxide (H₂O₂), which functions as a key signaling molecule, is essential for wound healing and activation of the regenerative response (47). FGF signaling positively interacts with the Sonic Hedgehog pathway through the *shh* gene (40). Recent evidence indicates a reciprocal relationship between *shh* signaling and the activity of superoxide dismutase (SOD), an enzyme that generates H₂O₂ from superoxide radicals (43). Pharmacological inhibition of s*hh* leads to defects in caudal fin regeneration associated with impaired proliferation and differentiation during tissue outgrowth (40), whereas disruption of H₂O₂ signaling also compromises regeneration (45). Comparable defects occur when BMP signaling is inhibited (48).

To determine whether AV gel influences these regeneration-related pathways, we quantified the expression of *the fgf20, bmp4*, and *shha* genes in the larval tail region at 6 and 24 hpa. Except for *fgf20,* pre-AV treatments significantly upregulated these genes during the early wound-healing phase, and expression levels returned close to control levels by 24 hpa. The exceptions wer*e bmp4* in the pre-AV group and *shha* in the post-AV group; these differences may reflect regulatory interactions among these pathways and the distinct timing of exposure to AV gel.

These results show that AV gel positively modulates key signaling pathways involved in regeneration, particularly those governing cell proliferation, differentiation, and tissue patterning. This molecular response may contribute to the increased regenerated area observed in the preAV and pre + postAV treatment groups at 5 dpa. This effect was not replicated in the postAV group, indicating that AV added to the zebrafish medium exerted a regenerative effect only when administered before caudal fin damage. The mechanisms underlying this effect should be elucidated in further experiments to provide a more detailed understanding of how AV treatment improves cell proliferation during regeneration.

Immediately after injury, inflammatory cells are rapidly recruited to the wound site. Damaged cells release H₂O₂, which acts as a chemoattractant for leukocytes and initiates the acute inflammatory phase required for effective healing. Neutrophils are the first responders, arriving within minutes and releasing myeloperoxidase (Mpx), an enzyme that degrades H₂O₂ and thereby contributes to resolution of the leukocyte-recruitment signal (49). A related inflammatory mediator is cyclooxygenase-2 (Cox-2), encoded by the *cox2* gene and expressed in epidermal and inflammatory cells in response to pro-inflammatory cytokines such as interleukin-1β (IL-1β). Cox-2 plays an important role in regeneration through prostaglandin synthesis and has been associated with wound closure, angiogenesis, and muscle growth (50).

Macrophages arrive later at the injury site to clear cellular debris, enabling tissue proliferation and regeneration. Together with epidermal cells, macrophages also produce IL-1β, a pro-inflammatory cytokine required for fin-fold regeneration in zebrafish. However, sustained IL-1β expression beyond 12–24 h can induce apoptosis and impair regeneration (51). Accordingly, our results show significant increases in the expression of *il-1b* and *cox-2* and the neutrophil and macrophage markers *mp*x and *mpeg1* during the acute inflammatory phase in the AV-treated groups; this finding was confirmed by increased neutrophil recruitment. Previous studies have highlighted the essential roles of macrophages and IL-1β signaling in zebrafish fin-fold regeneration, as well as the importance of regulating ROS levels in the wound microenvironment. Interestingly, ROS and IL-1β have been proposed to act as molecular links connecting the initial injury response with activation of regenerative pathways (19).

Consistent with this framework, our results show that AV administration increased neutrophil recruitment to the wound site at this stage. Importantly, immune-related gene expression and *in vivo* neutrophil counts *m*easured using the Tg(*lysC: D*sRED2) transgenic reporter line returned to control levels by 24 hpa (22, 23, 52). These results corroborate that AV exerts an immunomodulatory effect that enhances the early inflammatory phase while allowing its timely resolution.

Our molecular analyses also confirmed the antioxidant effects of AV during regeneration. Tail tissues showed a marked increase in the expression of the antioxidant genes *gpx, sod1*, and *sod2* at 6 hpa in all AV-treated groups. As observed for the immune genes, expression levels returned to near-control values by 24 h in most treatments, except in the group exposed to AV after amputation, confirming that this response was directly related to AV exposure.

These results confirm at the molecular level the rapid and significant immunostimulatory, antioxidant, and pro-regenerative effects of AV gel. Gene-expression changes were quantified in the excised wounded tail region of zebrafish larvae rather than in whole larvae. This approach allowed us to detect changes specifically in regenerating tissue during different phases of the process while avoiding interference from responses in other larval tissues.

### 4.2 *Aloe vera* mitigates the negative effects of an inflammatory diet on regeneration, growth, and mucus production

Pharmacological solutions commonly used to treat health problems in farmed fish can be labor-intensive when applied topically or by bath administration; therefore, delivering drugs or additives in the diet may be advantageous. Under farming conditions, fish are exposed to multiple stressors in addition to parasites, including antinutritional factors in the diet (53).

Previous studies in zebrafish have demonstrated that AV can counteract prolonged inflammation induced by soybean meal (SBM) diets and copper exposure, reducing neutrophil recruitment to the intestine and neuromast regions, respectively (31).

In the present study, we show that an SBM-based diet negatively affects tail-tip tissue regeneration and slows body growth in zebrafish larvae. Reduced growth is likely linked to intestinal inflammation and impaired nutrient absorption caused by antinutritional compounds in SBM (53). Importantly, supplementation of the SBM diet with AV increased acute neutrophil recruitment to the wound site and effectively reduced the negative effects of SBM on tail regeneration. Although macrophages were not directly visualized *in vivo* in this assay, their recruitment can be inferred from the increased transcript levels of the macrophage marker gene *mpeg1* in the tail region.

Taken together, the results presented here reinforce the idea that enhancement of the acute inflammatory response required during the early wound-healing phase, together with the antioxidant activity of AV, contributes to the improved regenerative capacity observed in zebrafish.

### 4.3 *Aloe vera* enhances mucus production and body growth in zebrafish larvae

Mucus is a critical component of immune defense in epithelial tissues directly exposed to the external environment, such as the skin, gills, and gastrointestinal tract. This barrier prevents parasites, bacteria, and fungi from penetrating deeper tissues while simultaneously supporting beneficial microbial communities. In this context, polysaccharides in *Aloe vera* gel, such as acemannans, have been reported to exert prebiotic effects that may benefit the microbiota (30). The primary structural components of mucus are high-molecular-weight glycoproteins known as mucins. Together with other molecules, including complement proteins, lysozymes, proteases, and antimicrobial peptides, mucins determine the protective and adhesive properties of mucus and regulate interactions with microorganisms. Disruption of mucus production or composition is associated with several intestinal and skin diseases and increased susceptibility to pathogens.

In fish, stressful conditions are known to alter mucus production and composition, resulting in reduced protective capacity. Therefore, dietary supplementation with natural compounds capable of enhancing immune responses and skin mucus production and promoting epithelial restoration may provide significant benefits (54, 55).

Fehrmann-Cartes et al. (33) reported that an SBM diet increased the number of goblet cells while downregulating expression of the *muc2.2* gene in the intestine of *Salmo salar*, whereas AV supplementation reversed both effects. These findings suggest that SBM reduces mucus production in fish intestines, whereas AV may help restore epithelial barrier function. In contrast, Hedrera et al. (21) observed no differences in intestinal goblet-cell numbers in zebrafish larvae after 4 days of feeding with SBM or fishmeal diets, possibly because of the relatively short exposure period or the limitations of conventional histological methods for accurately measuring cell size and quantifying cell numbers in extensive three-dimensional structures such as the intestine and skin.

To overcome these limitations, we used whole-larva Alcian Blue staining to detect acidic mucins produced by goblet cells. This technique allowed us to visualize and quantify the size and number of skin goblet cells within a defined area of the trunk region. Larvae fed the control diet supplemented with AV (FM + AV) exhibited increased goblet-cell size without a change in cell number. In contrast, the SBM diet significantly reduced the number of skin goblet cells and slightly decreased their size, although the latter effect was not statistically significant. Notably, supplementation of the SBM diet with AV (SBM + AV) significantly increased both the number and size of goblet cells. These findings differ from observations in the salmon intestine but suggest that dietary inclusion of *Aloe vera* can enhance mucus production in zebrafish larval skin, strengthening this critical physical and antimicrobial barrier against environmental threats and potentially supporting tissue regeneration following epithelial injury.

Taken together, our findings indicate that *Aloe vera* gel exerts a multifaceted immunomodulatory effect in zebrafish.

Although studies evaluating *Aloe vera* in fish remain limited, the available evidence, together with the findings of the present study, suggests that both topical administration through the aquatic environment and oral supplementation via the diet can modulate the innate immune response by influencing both inflammatory and antioxidant pathways during the early stages of injury, ultimately promoting improved tissue regeneration (56, 57). These effects are likely attributable to the combined action of the diverse bioactive compounds present in *Aloe vera* gel, which may act synergistically and additively (12–14). Among these compounds are anthraquinones, known to reduce oxidative stress and reactive oxygen species (ROS) production; antioxidant vitamins, including retinol; and polysaccharides, particularly acemannan, which exhibit prebiotic activity and contribute to maintaining intestinal homeostasis (58, 59).

Further research is needed to elucidate the specific molecular and cellular mechanisms underlying these beneficial effects on tissue regeneration and overall fish health. A deeper understanding of these processes could support the development of innovative strategies to enhance animal health and growth, thereby contributing to more sustainable aquaculture practices and reducing the environmental impact associated with intensive production systems.

## 5 Conclusion

This study demonstrates that *Aloe vera* gel enhances immune responses, antioxidant defenses, and tissue regeneration in zebrafish larvae following caudal injury. Although both immersion and dietary administration promoted regenerative responses, they appeared to exert complementary rather than identical effects. Immersion primarily enhanced the early inflammatory and antioxidant responses associated with wound healing, whereas dietary supplementation additionally improved larval growth, restored regeneration impaired by an inflammatory soybean meal-based diet, and increased skin goblet cell abundance, suggesting a broader role in maintaining mucosal health.

These findings support the potential of *Aloe vera* as a natural functional additive for improving fish health and welfare in aquaculture. However, this study was performed in zebrafish larvae, a well-established experimental model that enables rapid mechanistic evaluation but does not fully reproduce the physiological complexity or production conditions of commercially farmed fish. Therefore, although the conserved innate immune and regenerative pathways among teleosts support the translational value of these findings, their application to salmonids and other aquaculture species should be validated under species-specific farming conditions. Future studies should evaluate optimal administration strategies, dosage, long-term effects, and efficacy under commercial production settings.

## Data Availability

The original contributions presented in the study are included in the article/supplementary material, further inquiries can be directed to the corresponding author/s.
